# The burden, admission, and outcomes of COVID-19 among asthmatic patients in Africa: protocol for a systematic review and meta-analysis

**DOI:** 10.1186/s40733-020-00061-x

**Published:** 2020-09-04

**Authors:** Abrha Hailay, Woldu Aberhe, Kidane Zereabruk, Guesh Mebrahtom, Teklehaimanot Haile, Degena Bahrey, Teklewoini Mariye

**Affiliations:** 1 Department of Adult health Nursing, School of Nursing, Aksum University, Aksum, Ethiopia; 2 Department of Maternity and Neonatal Nursing, School of Nursing, Aksum University, Aksum, Ethiopia

**Keywords:** Admission, Asthma, Africa, Burden, COVID-19, Outcome

## Abstract

**Background:**

Coronavirus disease 2019 outbreak is the first reported case in Wuhan, China in December 2019 and suddenly became a major global health concern. According to the European Centre for Disease Prevention and Control, on August 4, 2020 the reported cases of coronavirus disease 2019 were 18,456,952 cases worldwide, 11,691,229 recovered with 697,719 deaths. Evidence on Burden, admission and outcome of Coronavirus Disease in 2019 among Asthmatic patients has not been published in Africa. This research protocol will, therefore, be driven to conduct systematic review and meta-analysis of the Coronavirus Disease in 2019 burden, admission and outcome among Asthmatic patients in Africa.

**Methods:**

All observational studies among Asthmatic patients in Africa and written without language limitation will be included. A search technique was applied using Databases (PubMed / MEDLINE, EMBASE, HINARI, Cochrane Library, World Health Organization COVID-19 database, Africa Wide Knowledge and Web of Science). Two independent authors carried out data extraction and assess the risk of bias using a predetermined and structured method of data collection. We will use random-effects to estimate the overall pooled burden, admission and outcome of COVID-19 Asthmatic patients in Africa. To assess possible publication bias, funnel plot test and Egger’s test methods will be used**.** This systematic and meta-analysis review protocol will be reported based on the Preferred Reporting Items for Systematic reviews and Meta-Analysis protocol guidelines.

**Discussion:**

The description will be used to show the COVID-19 distribution data by interest variables such as residence, setting, and person-level characteristics. The findings of this review will notify health care professionals about the burden, admission and outcome of COVID − 19 in asthmatic patient, while providing evidence to bring about the requisite improvements in clinical practice for asthmatic patients.

**Systematic review registration:**

This review is registered in the PROSPERO International Prospective Register of Systematic reviews with the registration number of CRD42020202049.

## Background

In December 2019, a new virus (initially called ‘Novel Coronavirus 2019-nCoV’ and later renamed to SARS-CoV-2) causing severe acute respiratory syndrome (coronavirus disease COVID-19) emerged in Wuhan, Hubei Province, China, and rapidly spread to other parts of China and other countries around the world and it suddenly became a major global health concern [1]. The World Health Organization (WHO) has called the outbreak of coronavirus disease 2019 (COVID-19) a global emergency [2].

COVID-19’s clinical symptoms vary from asymptomatic illness to flu-like disease, including high morbidity and mortality from multi-organ failures. The majority of patients diagnosed with COVID-19 have developed mild symptoms including sore throat, dry cough, and fever. The majority of them have resolved spontaneously. Some have developed multiple fatal complications, such as: septic shock, extreme pneumonia, and organ failure [3].

According to the European Centre for Disease Prevention and Control, on August 4, 2020, the reported cases of coronavirus disease 2019 were 18,456,952 cases worldwide, 11,691,229 recovered with 697,719 deaths. The United States has the largest number of confirmed cases 4,862,285 cases, 2,447,525 recovered with 158,931 deaths in the world and followed by Brazil has 2,751,665cases, 1,912,319 recovered and with 94,702 deaths. In Africa, the total number of confirmed were 972,374 cases, 633,289recovered and 20,682 deaths. South Africa has the largest number of reported 511,485 cases with 8366 total deaths cases in Africa [1, 4].

According to different Studies, clinical features of COVID-19 infection is varied based on the groups and showed greater risks for the development of pneumonia as a serious type of infection among the elderly people and with chronic comorbidities, especially systemic arterial hypertension, diabetes mellitus and immunosuppression. Additionally, official Chinese information showed that 8% of pregnant women with COVID-19 were serious cases and may also be especially susceptible to infection [5–8]. Patients with asthma have likely to develop severe COVID-19 and other complications than patients without Asthma [9].

There have been different reports about COVID-19 [10–13]. Forty-five percent (45%) patients with COVID − 19 receive non-invasive respiratory support via a non-rebreathing oxygen face mask [14]. Sputum cells among patients with asthma may give a risk for COVID-19 morbidity [15]. The prevalence of COVID-19 among asthmatic patient was 9% [16]. Yet, there is no pooled result of the COVID-19 burden, admission and outcome among asthmatic patients in Africa. This research protocol will, therefore, be driven to conduct systematic review and meta-analysis of the COVID-19 burden, admission and outcome among asthmatic patients in Africa.

## Methods

### Protocol registration

This review is registered in the PROSPERO International Prospective Registry of Systematic Reviews (CRD42020202049) and reported according to Preferred Reporting Items for Systematic reviews and Meta-Analysis protocol (PRISMA-P) guidelines [17] (Table 1).

**Table 1:** PRISMA-P 2015 checklist: recommended items to address in a systematic review protocol

Section/topic	Item No	Checklist item	Information reported		Line number(s)
			Yes	No	
Administrative Information					
Title					
Identification	1a	Identify the report as a protocol of a systematic review	☐	☐	
Update	1b	If the protocol is for an update of a previous systematic review, identify as such	☐	☐	
Registration	2	If registered, provide the name of the registry (e.g., PROSPERO) and registration number in the Abstract	☐	☐	
Authors					
Contact	3a	Provide name, institutional affiliation, and e-mail address of all protocol authors; provide physical mailing address of corresponding author	☐	☐	
Contributions	3b	Describe contributions of protocol authors and identify the guarantor of the review	☐	☐	
Amendments	4	If the protocol represents an amendment of a previously completed or published protocol, identify as such and list changes; otherwise, state plan for documenting important protocol amendments	☐	☐	
Support					
Sources	5a	Indicate sources of financial or other support for the review	☐	☐	
Sponsor	5b	Provide name for the review funder and/or sponsor	☐	☐	
Role of sponsor/funder	5c	Describe roles of funder(s), sponsor(s), and/or institution(s), if any, in developing the protocol	☐	☐	
Introduction					
Rationale	6	Describe the rationale for the review in the context of what is already known	☐	☐	
Objectives	7	Provide an explicit statement of the question(s) the review will address with reference to participants, interventions, comparators, and outcomes (PICO)	☐	☐	
Methods					
Eligibility criteria	8	Specify the study characteristics (e.g., PICO, study design, setting, time frame) and report characteristics (e.g., years considered, language, publication status) to be used as criteria for eligibility for the review	☐	☐	
Information sources	9	Describe all intended information sources (e.g., electronic databases, contact with study authors, trial registers, or other grey literature sources) with planned dates of coverage	☐	☐	
Search strategy	10	Present draft of search strategy to be used for at least one electronic database, including planned limits, such that it could be repeated	☐	☐	
Study Records					
Data management	11a	Describe the mechanism(s) that will be used to manage records and data throughout the review	☐	☐	
Selection process	11b	State the process that will be used for selecting studies (e.g., two independent reviewers) through each phase of the review (i.e., screening, eligibility, and inclusion in meta-analysis)	☐	☐	
Data collection process	11c	Describe planned method of extracting data from reports (e.g., piloting forms, done independently, in duplicate), any processes for obtaining and confirming data from investigators	☐	☐	
Data items	12	List and define all variables for which data will be sought (e.g., PICO items, funding sources), any pre-planned data assumptions and simplifications	☐	☐	
Outcomes and prioritization	13	List and define all outcomes for which data will be sought, including prioritization of main and additional outcomes, with rationale	☐	☐	
Risk of bias in individual studies	14	Describe anticipated methods for assessing risk of bias of individual studies, including whether this will be done at the outcome or study level, or both; state how this information will be used in data synthesis	☐	☐	
*Data*					
Synthesis	15a	Describe criteria under which study data will be quantitatively synthesized	☐	☐	
	15b	If data are appropriate for quantitative synthesis, describe planned summary measures, methods of handling data, and methods of combining data from studies, including any planned exploration of consistency (e.g., *I* ^2^, Kendall’s tau)	☐	☐	
	15c	Describe any proposed additional analyses (e.g., sensitivity or subgroup analyses, meta-regression)	☐	☐	
	15d	If quantitative synthesis is not appropriate, describe the type of summary planned	☐	☐	
Meta-bias (es)	16	Specify any planned assessment of meta-bias (es) (e.g., publication bias across studies, selective reporting within studies)	☐	☐	
Confidence in cumulative evidence	17	Describe how the strength of the body of evidence will be assessed (e.g., GRADE)	☐	☐	

### Eligibility criteria

#### Types of study

All observational studies; including cross-sectional studies, cohort, case-control, and baseline results from randomized controlled trials carried out in Africa will be included.

#### Participants/population

All Asthmatic patients (all studies include all age groups) who are African residence and laboratory-confirmed and/or clinically diagnosed with having COVID-19.

#### Intervention(s), exposure(s)

Asthma with COVID-19 infection. Therefore, we want to assess disease burden, admission and outcome of COVID-19 on asthmatic patients will be reviewed.

#### Outcomes

Morbidity, admission, Mortality and other clinical outcomes of COVID-19 among asthmatic patients (Prevalence rate, infection rate Clinical characteristics include symptoms (such as symptoms of upper respiratory tract infection, myalgia, fever, cough, dyspnea/shortness of breath, electrolyte imbalance and organ failure). laboratory/imaging (chest X-ray, CT, C-Reactive protein and whole blood count), and outcomes of Asthma (recovery, complications and death) and COVID-19 outcomes.

#### Settings

Hospital-based studies.

#### Language

Without the restriction of language all published and unpublished papers will be included in this review.

#### Method of diagnosis

No limitation on diagnostic methods but subgroup review will be carried out based on diagnostic instruments. Interim guidance from the WHO and/or any diagnostic criteria proposed by the WHO shall be considered ‘WHO interim guidance for laboratory biosafety related to 2019-nCoV’ [18, 19] (Table 2).

**Table 2:** Laboratory examination of coronavirus disease in suspected human cases: interim guidance

Test	Type of sample	Timing
Nucleic Acid Amplification Tests (NAAT)	**Upper respiratory specimens:** nasopharyngeal and oropharyngeal swab nasopharyngeal wash/nasopharyngeal aspirate **Lower respiratory specimens:** sputum, endotracheal aspirate or bronchoalveolar lavage are preferred for patients with severe respiratory disease.	Upon introduction pick. Maybe repeated sampling to clearance monitor. Additional work is required to assess the repeated sampling is for efficient and accurate.
Serology and other	blood and stool are also responsible for the coronaviruses (COVID-19)	Paired samples are needed to confirm with the original sample obtained during disease of first week and the second one preferably obtained after 2–4 weeks (There has to be an ideal timing for convalescent samples Undertaken).

#### Exclusion criteria

Studies that have not clarified the requirements for the COVID-19 outcome level; studies that have not been conducted in humans, qualitative studies, studies that lack valid data required to determine the outcome will not be included. Studies such as experimental studies, commentaries, editorials, letters, case reports, or case series will be excluded from this review.

### Search strategy and data source

A search technique was applied using online Databases (PubMed / MEDLINE, EMBASE, HINARI, Cochrane Library, WHO COVID-19 database, Africa Wide Knowledge and Web of Science) from February to August 2020 (Table 3). The quest was performed either individually or in combination using the following keywords: Admission, Asthma, burden, COVID-19, outcome and prevalence. Search terms to be used: “Wuhan coronavirus” OR “COVID-19” OR “novel coronavirus” OR “2019-nCoV” OR “Coronavirus outbreak” OR “SARS-CoV-2” OR “SARS2” OR “Severe acute respiratory syndrome coronavirus 2” OR “Burden” OR “Outcome”. Other searching terms will be “mortality” OR “prevalence” OR “incidence” OR “Asthma complication of COVID-19”.

**Table 3:** Searching strategy

S.No	Databases	Number of articles found	Number of articles included	Number of articles excluded	Reason for exclusion
1	Google Scholar	N=	N=	N=	
2	PubMed / MEDLINE	N=	N=	N=	
3	EMBASE	N=	N=	N=	
4	HINARI	N=	N=	N=	
5	Cochrane Library	N=	N=	N=	
6	WHO COVID-19 database	N=	N=	N=	
7	Africa Wide Knowledge	N=	N=	N=	
8	Web of Science	N=	N=	N=	
9	Unpublished thesis, manuscript, and report from WHO and CDC	N=	N=	N=	

### Selection and data collection process

Data were extracted using a standardized method of data extraction. Two assessors (AH and WA) will autonomously extract data using the predefined standardized extraction form from the included studies. For further consideration of whether to include in the study or not, full texts for the qualifying titles and/or abstracts, including those where there is ambiguity, will be collected. The agreement between the reviewers of the study will be calculated using Cohen’s λ statistics. Disagreements will be resolved by mediation, and arbitration by a third reviewer (GM) will occur when necessary. Reasons for excluding articles will be noted.

Where there is missing information, authors have been contacted for additional details to ensure study eligibility. Where necessary, up to three emails have been sent to the corresponding author to request additional information before excluding the study. We will consider the most recent, detailed, and with the highest sample size for studies that appear in more than one published article. We shall treat each survey as a separate study for surveys that appear in one article with multiple surveys conducted at different time points. Data extraction was including information: first author, publishing month, country and/or region, signs and symptoms, complications, diagnostic criteria, comorbidity, COVID-19, study area, prevalence and/or incidence, characteristics of the study (study design, response rate).

### Quality assessment and risk of bias in individual studies

A tool developed by Hoy et al. for prevalence studies will be used to evaluate the likelihood of bias and quality of studies included in this review [20]. The tool contains 11 items; items 1–4 assess the external validity, 5–10 assess the internal validity, and item 11 offers a description of the overall risk by the reviewer based on the responses of the above 10 items which are rated 1 if yes and 0 if no. Studies are graded as low (< 3), moderate risk (4-6) or high (7-9) risk of bias. Two reviewers did this exercise, and disputes will be resolved through discussion and, where possible, through arbitration involving a third author. Besides, adequate sampling methods, consistent methods and procedures for collecting data, recorded methods of quality control and representative sample size will be considered as indicators of the study quality. Studies of high quality will be studies that revealed all the points mentioned above.

### Data management

A framework was developed a priori to guide the screening and selection process, based on the inclusion and exclusion criteria. The tool will be piloted and revised before data extraction begins. First, to delete duplicates, the search results will be uploaded to EndNote software. The remaining articles will be put on Rayyan, a smartphone and a web-based software system that facilitates the collaboration between reviewers involved in the screening and selection of studies to be included in the review [21].

### Data items

Data extraction was including: authors, month, country and/or region, sample size, type of publication, study area, characteristics of the study (study design, response rate).

### Outcomes and prioritization

The primary outcome is the burden, admission and outcome of COVID − 19 among asthmatic patients in African.

### Data analysis and presentation of results

R software and R studio will be used during analyzing the Data. All analyses will be carried out using a “metaprop” routine for Windows using R version 3.5.3 [22]. Results will be reported as proportions with corresponding 95% confidence intervals (CIs). Forest plots will be drawn to represent the combined outcome of COVID-19 and the extent of statistical heterogeneity among studies. The statistical heterogeneity will be evaluated using the χ2 test and quantified using the calculation of the I^2^ statistics with values of 25, 50 and 75% being representative of low, medium and high heterogeneity, respectively [23]. If there will be heterogeneity between studies, we will use a meta-analysis of random-effects [24] to estimate the aggregate pooled burden, admission and outcome of COVID-19 among asthmatic patients in Africa. To assess possible publication bias, funnel plot test and Egger’s test methods will be used [25]. *P*-value < 0.10 on the Egger’s test is considered statistically significant for bias in writing.

### Data synthesis

The study-specific outcome of COVID-19 among asthmatic patients will be recalculated using Crude numerators and denominators from individual studies. A meta-analysis will be performed on variables that are similar across the included studies. Because there will be heterogeneity among the studies, the random effect model will be used to determine the pooled burden, admission and outcome of COVID-19 in Africa. African Geographic regions, diagnostic methods, and based on their ethnic background where the study was conducted will be summarized by a subgroup analysis.

## Discussion

**Fig. 1:**
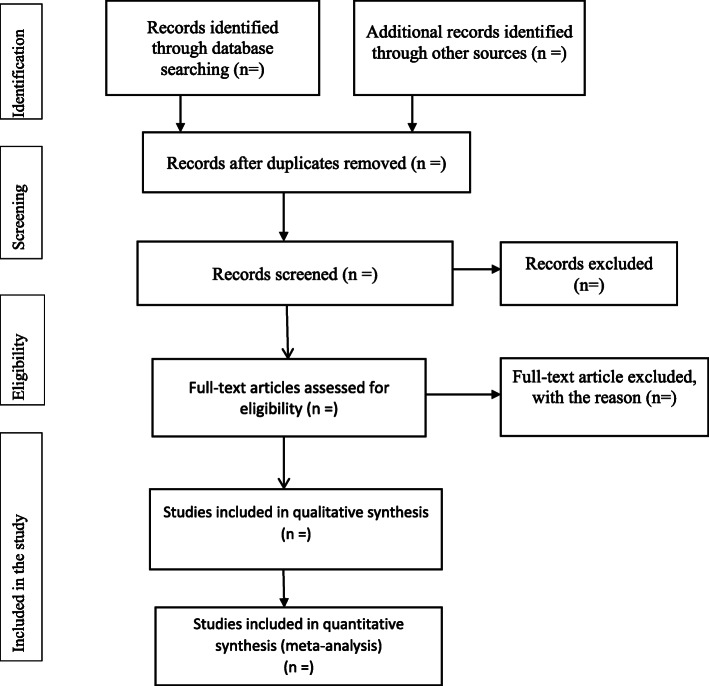
Selection of articles for systemic review and meta-analysis of burden, admission and 210 outcome of COVID-19 among Asthmatic patients in Africa

This review will be done based on the PRISMA-P guidelines and the PRISMA flow diagram and also used to document the different phases of the review process [17] (Fig. 1).

The findings of this review will notify to health program planners, decision-makers and health care professionals about the burden, admission and outcome of COVID − 19 among asthmatic patients, while providing evidence to bring good quality health care, the good emphasis for the problem, improvements in clinical practice. Conferences, peer-review articles, and social media sites will share conclusions from this study.

## Conclusion

This systematic review and meta-analysis will be expected to quantify the burden, admission and outcome of COVID-19 among asthmatic patients in Africa.

## Data Availability

This study has not been submitted and considered for publish in any journal. The datasets used and/or analyses during the study will be presented within the manuscript and available from the corresponding author on request.

## Abbreviations

COVID-19: Coronavirus Disease in 2019
PRISMA-P: Preferred Reporting Items for Systematic reviews and Meta-Analysis protocol
WHO: World Health Organization
